# Hospitalizations Associated with Disseminated Coccidioidomycosis, Arizona and California, USA

**DOI:** 10.3201/eid1809.120151

**Published:** 2012-09

**Authors:** Amy E. Seitz, D. Rebecca Prevots, Steven M. Holland

**Affiliations:** National Institutes of Health, Bethesda, Maryland, USA

**Keywords:** disseminated coccidioidomycosis, epidemiology, race, ethnicity, Arizona, California, United States, fungi, hospitalization

## Abstract

We analyzed hospitalization databases from Arizona and California for disseminated coccidioidomycosis–associated hospitalizations among immunocompetent persons. Racial/ethnic disease ratios were characterized by a higher incidence of hospitalization among blacks compared with other groups. This finding suggests that HIV infection, AIDS, and primary immune conditions are not a major factor in this disparity.

Coccidioidomycosis is a fungal infection caused by inhalation of *Coccidioides immitis* or *C. posadasii* spores (*1*,*2*). Most (≈60%) persons infected with *Coccidioides* spp. are asymptomatic (*3*), but symptomatic primary pulmonary coccidioidomycosis develops in ≈40% (*4*). An estimated 1% of all infections, asymptomatic and symptomatic, progress to extrathoracic disseminated coccidioidomycosis infection (*3*,*4*), which is more common among persons with underlying immune conditions, such as advanced HIV/AIDS. Although disseminated coccidioidomycosis is most often found in immunocompromised persons, it can also occur among persons without known predisposing conditions (*5*). The epidemiologic association between ethnicity and disseminated coccidioidomycosis within disease-endemic regions has been described (*6*,*7*) but not specifically among persons with disseminated coccidioidomycosis without HIV/AIDS or primary immune deficiencies. In the United States, coccidioidomycosis is predominantly localized to specific areas of southern California and Arizona (*8*). We focused our analyses of racial/ethnicy disease ratios on these regions.

## The Study

We used the Arizona and California State Inpatient Databases (SID) from the US Agency for Healthcare Research and Quality (AHRQ) to describe clinical and demographic characteristics of persons hospitalized for disseminated coccidioidomycosis who did not have known primary immune conditions, HIV infection, or AIDS. The SID contains state-level data, including information from 90% of community hospital in-patient stays, and is maintained as part of the Healthcare Utilization Project at AHRQ. We extracted records from the available annual datasets for Arizona (2000–2009) and California (2003–2008) that listed any diagnosis of disseminated coccidioidomycosis (code 114.3 from the International Classification of Diseases, 9th Revision, Clinical Modification [ICD-9-CM]). We excluded records that listed any diagnosis of HIV infection or AIDS (ICD-9-CM code 042) or primary immune deficiency (ICD-9-CM code 279.xx). This study was not considered human subject research by the National Institutes of Health Office of Human Subjects Protection.

We calculated the average annual incidence of hospitalizations as the average annual number of disseminated coccidioidomycosis hospitalizations divided by the midyear population, as determined by US Census estimates (*9*). By using the revisit files available from AHRQ, we assessed differences in readmissions by race by comparing the proportion of persons with only 1 disseminated coccidioidomycosis infection among race groups. We also calculated all-cause hospitalization rates for blacks and whites in our dataset by using total hospitalizations from HCUPnet (http://hcupnet.ahrq.gov). In years for which patient state of residency was available, we assessed the potential bias from hospitalizations of persons with out-of-state residency by determining the percentage of total admissions from out-of-state patients. We used the Mann-Whitney test for nonparametric comparisons; the significance level used for all statistical tests was α = 0.05. All analyses were completed in SAS version 9.2 software (SAS Institute, Cary, NC, USA).

**Figure 1:**
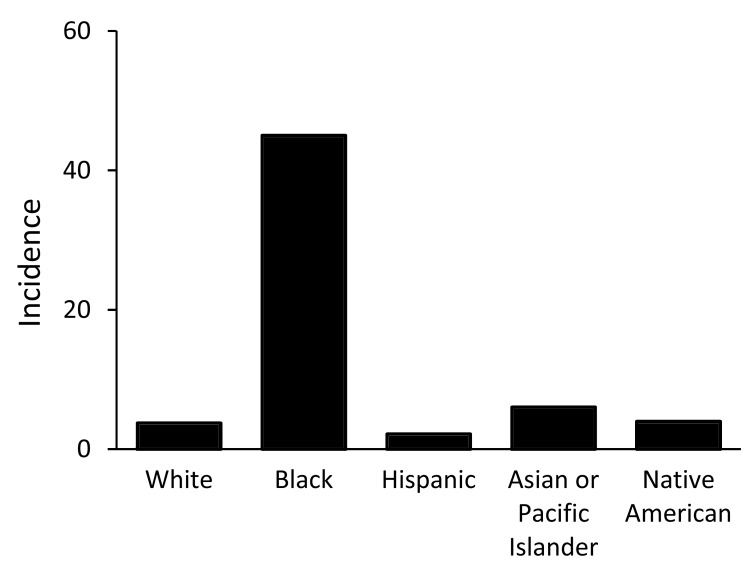
Average annual incidence (per 100,000 population) of disseminated coccidioidomycosis–associated hospitalizations, by race/ethnicity, Arizona, USA.

**Figure 2:**
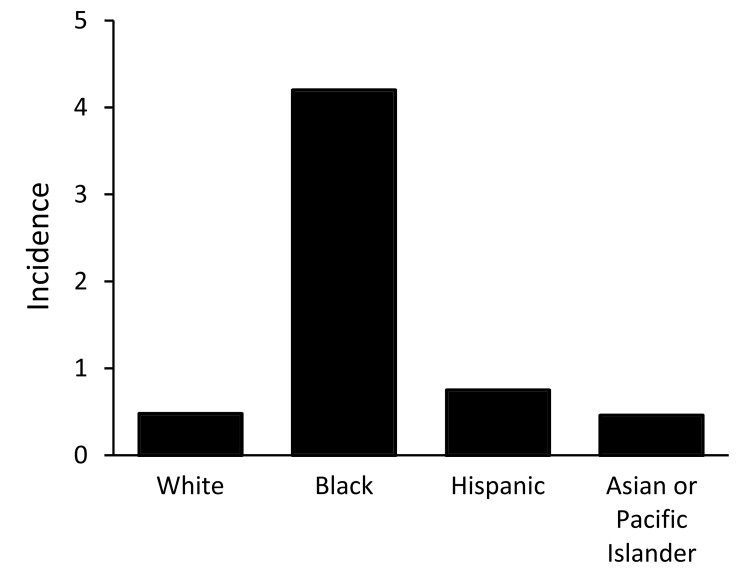
Average annual incidence (per 100,000 population) of disseminated coccidioidomycosis hospitalizations, by race/ethnicity, California, USA. (Note: Average annual incidence not reported for Native Americans because of low numbers.)

We identified 4,719 disseminated coccidioidomycosis–associated hospitalizations. The average annual incidence of hospitalizations/100,000 persons/year was 4.8 (95% CI 4.2–5.3) for Arizona and 0.89 (95% CI 0.79–0.99) for California. Overall, the rate of hospitalization for blacks in Arizona was 12.0-fold higher than the rate of hospitalization for whites and 20.8-fold higher than for Hispanics (Figure 1). The rate of hospitalization in California was 8.8-fold higher for blacks than for whites and 5.6-fold higher for blacks than for Hispanics (Figure 2).

The median age of patients with disseminated coccidioidomycosis–associated hospitalizations was lower than the median age of all hospitalizations for both blacks and whites in Arizona and California (p<0.001 for all comparisons). The median age at hospitalization for Hispanics was higher than the median age for all hospitalizations in Arizona (p<0.001) and California (p = 0.01) (Table).

**Table:** Characteristics of patients hospitalized for all causes and for disseminated coccidioidomycosis, by patient race/ethnicity and age, Arizona and California, USA*

Characteristic	Arizona hospitalizations			California hospitalizations	
	Disseminated coccidioidomycosis	All causes		Disseminated coccidioidomycosis	All causes
Total, no. patients	2,770	6,180,404		1,949	19,979,016
Race/ethnicity, no. (%) patients					
White	1,316 (47.5)	4,207,269 (68.1)		451 (23.1)	10,541,469 (52.8)
Black	771 (27.8)	219,265 (3.5)		561 (28.8)	1,520,896 (7.6)
Hispanic	360 (13.0)	1,240,707 (20.1)		587 (30.1)	4,957,476 (24.8)
Asian/Pacific Islander	80 (2.9)	76,345 (1.2)		125 (6.4)	1,269,150 (6.4)
Native American	102 (3.7)	197,181 (3.2)		NR	13,819 (0.07)
Other	65 (2.3)	98,975 (1.6)		18 (0.9)	342,786 (1.7)
Information missing	76 (2.7)	140,662 (2.3)		NR	1,333,420 (6.7)
Median patient age, y					
Overall†	**47**	54		**43**	54
White	**56**	60		**51**	62
Black	**37**	43		**40**	51
Hispanic	**41**	33		**41**	35
Asian	39	39		**50**	57
Native American	**46**	39		NR	47

*Boldface indicates a significant difference (p<0.05) compared with all-cause hospitalizations. NR, information not reported because cell size is <10 or to prevent calculation of a cell size <10. †Excludes hospitalized patients with missing race/ethnic information.

The proportion of persons with >1 hospital admission for disseminated coccidioidomycosis among blacks was higher than for whites in California in 2008 (29.8% vs. 17.3%) and in Arizona in 2007 (19.3% vs. 17.0%). Among all disseminated coccidioidomycosis–associated hospitalizations, 98% of hospitalizations in Arizona and 99% of hospitalizations in California were from in-state residents, indicating minimal bias from nonresidents. All-cause hospitalization rates did not differ greatly between whites and blacks: 121 hospitalizations/1,000 persons for whites and 104 hospitalizations/1,000 persons for blacks in Arizona; 125 hospitalizations/1,000 persons for whites and 143 hospitalizations/1,000 persons for blacks in California.

## Conclusions

We identified a higher incidence and lower patient age at hospitalization for disseminated coccidioidomycosis–associated hospitalizations among blacks in California and Arizona. We were unable to determine if the higher incidence of hospitalization was a result of environmental, host, or behavioral factors. However, this study suggests that HIV infection, AIDS, and primary immune conditions are not the main reason for the racial/ethnic disparity for hospitalizations associated with disseminated coccidioidomycosis.

Because most coccidioidomycosis cases in California occur in the San Joaquin Valley region (*6*) and 90% of the population of California lives outside this region (*10*), the California-specific incidence rate is likely to be an underrepresentation of the extent of the disease in the San Joaquin Valley region. In addition, because of this disparity, we are unable to make comparisons between rates for California and Arizona. The state-specific incidence is a better description of the extent of disease in Arizona because the 3 counties with 93% of the coccidioidomycosis cases in Arizona represent 79% of the state’s population (*11*).

The classification of race/ethnicity in this dataset may not completely describe the true distribution of disease among the diverse groups comprising these populations. These categories include a wide range of racial/ethnic backgrounds, representing multiple potential environmental, social, cultural, behavioral, or genetic susceptibilities. However, the higher incidence among blacks suggests that unknown factors uniquely affect a high proportion of this population.

Our analysis is likely to be specific for accurately detecting the number of cases of disseminated coccidioidomycosis because most cases require hospitalization of the patient. Furthermore, in Arizona and southern California, where the infection is common, awareness of coccidioidomycosis and disseminated coccidioidomycosis is high, and disseminated coccidioidomycosis cases are likely to be recognized. This analysis method is also likely to be specific because results of cultures, biopsies, histologic testing, and serologic testing provide strong evidence of infection. However, our use of administrative data, such as the SID, is limited by the use of ICD-9-CM codes, and the sensitivity and specificity of those codes for disseminated coccidioidomycosis has not been evaluated.

An additional limitation of our study was that we could not determine if a single person was hospitalized multiple times during the study years. Although we identified higher rates of readmission among blacks, consistent with previous studies for coccidioidomycosis (*12*), this does not completely explain the large relative rates that we identified. This finding could indicate different disease pathology with more serious or long-term infection.

Potential bias from out-of-state residents was minimal because most disseminated coccidioidomycosis hospitalizations occurred within the state of residence. Differences in all-cause hospitalization rates were not likely to account for the differences in rates observed for our study condition. A better understanding of the progression of disease, including the number of previous hospitalizations, information on all coexisting conditions, and the severity of disease, could help explain the differences in incidence of hospitalization.

Overall, we found a higher incidence of disseminated coccidioidomycosis–associated hospitalizations for blacks compared with whites and other racial/ethnic groups living in these coccidioidomycosis-endemic areas, a finding that is consistent with previous studies (*6*,*7*). However, our study identifies this difference specifically in the absence of HIV/AIDS and primary immune conditions among a large cohort, which suggests other, unknown reasons for this disparity among races/ethnicities.
